# Milk Odd- and Branched-Chain Fatty Acids as Biomarkers of Rumen Fermentation

**DOI:** 10.3390/ani14111706

**Published:** 2024-06-06

**Authors:** Robert Kupczyński, Katarzyna Pacyga, Kamila Lewandowska, Michał Bednarski, Antoni Szumny

**Affiliations:** 1 Department of Environment Hygiene and Animal Welfare, The Faculty of Biology and Animal Science, Wroclaw University of Environmental and Life Sciences, 38c Chelmonskiego St., 50-375 Wroclaw, Poland; katarzyna.pacyga@upwr.edu.pl (K.P.); kamila.lewandowska@upwr.edu.pl (K.L.); 2 Department of Epizootiology and Clinic of Bird and Exotic Animals, Faculty of Veterinary Medicine, Wroclaw University of Environmental and Life Science, 47 Grunwaldzki Sq., 50-366 Wroclaw, Poland; michal.bednarski@upwr.edu.pl; 3 Department of Food Chemistry and Biocatalysis, Wrocław University of Environmental and Life Sciences, 50-375 Wroclaw, Poland; antoni.szumny@upwr.edu.pl

**Keywords:** fatty acids, branched-chain fatty acids, biomarker, *iso* fatty acid

## Abstract

**Simple Summary:**

In recent years, research has focused on determining the contents of odd- and branched-chain fatty acids (OBCFAs) in milk from various ruminant species, examining the relationship between rumen fermentation processes and changes in feed composition. The content of OBCFAs in cow’s milk fat depends on the composition of the rumen microbiota, influenced by factors such as the feeding system, feed composition, proportion of roughage to concentrate, and rumen content pH. Therefore, the profiling of FAs in milk is of the utmost importance, as they can be considered as a noninvasive biomarker for predicting the proportions of volatile fatty acids (VFAs) in the rumen, microbial protein synthesis, or susceptibility to ruminal acidosis in dairy cows. High-fiber diets generally have a lower milk OBCFA content than lower-fiber diets. Some OBCFAs found in milk fat can be markers of ruminal acidosis or a negative energy balance. In this review, we present an up-to-date summary of research on the role and significance of OBCFAs in rumen fermentation processes and the link between these relationships and the potential for diagnosing metabolic disorders in dairy cows.

**Abstract:**

Cow’s milk and dairy products are the primary sources of OBCFAs, which have beneficial health properties. The goal of this study was to identify the factors that influence the content of OBCFAs in cow’s milk and to indicate which OBCFAs can serve as biomarkers for fermentation processes. The content of OBCFAs in milk depends on the species of ruminants, with studies showing that this varies between 3.33% (in goat’s milk) and 5.02% (in buffalo’s milk). These differences also stem from the animals’ energy balance, lactation phases, forage-to-concentrate ratio, and the presence of bioactive compounds in feeds, as well as management practices and environmental conditions. The OBCFAs in milk fat mainly come from rumen bacteria, but can also be synthesized de novo in the mammary gland, making them potentially useful noninvasive indicators of rumen fermentation. The concentration of BCFA is lower in colostrum and transitional milk than in full lactation milk. The proportions of total OBCFAs are higher in first- and second-parity cows. The most effective predictors of the biohydrogenation of fatty acids in the rumen are likely C18:2 *cis*-9, *trans*-11, *iso*-C16:0, and *iso*-C13:0. OBCFAs have been identified as potential biomarkers for rumen function, because their synthesis depends on specific bacteria. Strong predictors of subclinical ruminal acidosis include *iso*-C14:0, *iso*-C13:0, and C15:0. The concentration of ∑ OBCFA >C16 in milk is associated with fat mobilization and serves as a significant marker of the energy balance in cows.

## 1. Introduction

**Figure 1:**
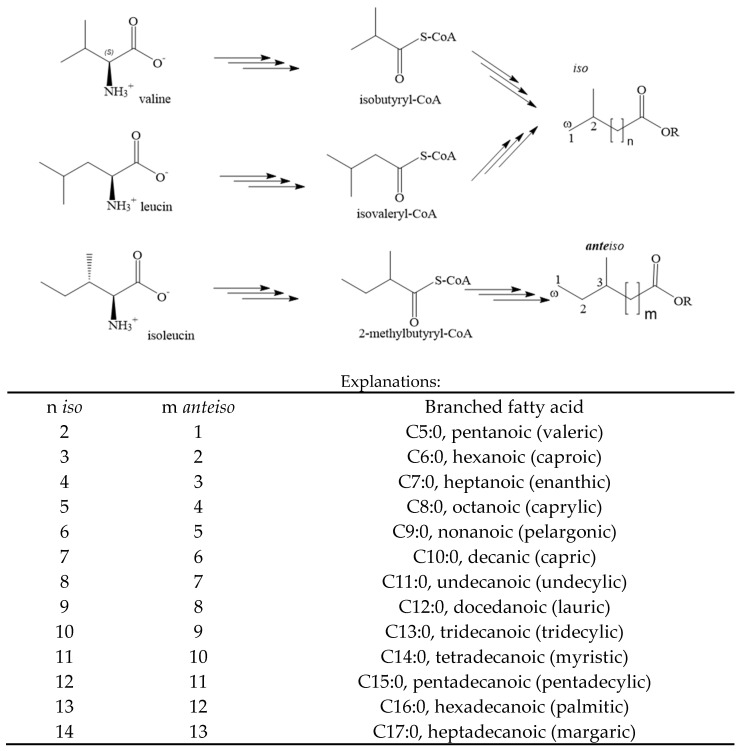
Biosynthesis and structures of main branch-chained fatty acids.

Milk and dairy products are excellent sources of protein and minerals, as well as a valuable source of fat. A characteristic feature of milk fat is its high digestibility, resulting from the fine dispersion of fat globules, allowing for easier digestion. The first step of this process is the partial hydrolysis of the milk fat globules by gastric lipases, initiated in the stomach, whereas the final hydrolysis occurs in the duodenum by pancreatic lipases [1]. Milk fat has a complex composition of fatty acids with varying numbers of carbon atoms and different degrees of unsaturation. Many of these fatty acids have specific biological and nutritional properties, valuable for young organisms (e.g., calves), as well as for human health [2,3,4,5]. The least understood group of acids are odd- and branched-chain fatty acids, abbreviated as OBCFAs. This group includes specific fatty acids with an odd number of carbon atoms, as well as those with a methyl group placed on the C2 (*iso*) or C3 (*anteiso*) carbon from the methyl end (ω)-carbon of the chain (Figure 1). Translated from Latin, the prefix “*ante*” literally means “before in front”, which precisely describes the location of the branch methyl group in the structure.

Although the OBCFAs in cow’s milk fat can consist of chains ranging in length from C3 to C27, research on ruminants typically focuses on isomers of tetradecanoic acid (*iso*-C14:0, n = 11), pentadecanoic acid (C15:0, *iso*-C15:0, n = 12, and *anteiso*-C15:0, m = 11), hexadecanoic acid (*iso*-C16:0, n = 13), and heptadecanoic acid (C17:0, *iso*-C17:0, n = 14, and *anteiso*-C17:0, m = 13). Quantitative and qualitative changes in rumen bacteria lead to a change in the profile of the OBCFAs in milk fat. The OBCFA content in cow’s milk fat is largely the result of bacterial processes in the rumen [3,6], although the endogenous synthesis and/or conversion of certain acids following lipomobilization also plays a role. The cell membrane lipids of rumen bacteria contain a significant amount of OBCFAs, including pentadecanoic acid (C15:0), *iso* methyltetradecanoic acid (*iso*-C15:0), *anteiso* methyltetradecanoic acid (*anteiso*-C15:0), heptadecanoic acid (C17:0), *iso* methylhexadecanoic acid (*iso*-C17:0), *anteiso* methylhexadecanoic acid (*anteiso*-C17:0), and heptadecenoic acid (C17:1) [7]. Branched-chain amino acids (valine, leucine, and *iso*leucine) are precursors to branched-chain fatty acids (*iso*-C15:0, *iso*-C17:0, *anteiso*-C15:0, and *anteiso*-C17:0) and their branched-short-chain carboxylic acids (*iso*butyric, *iso*valeric, and 2-methylbutyric acids) [3,7]. The rumen contains a rich population of bacteria with specific enzymes responsible for the de novo synthesis of membrane fatty acids [6]. Thus, the content and proportions of OBCFAs reflect changes in the rumen bacterial populations, and, at the same time, they indicate the functioning of fermentation processes in the rumen. Recent studies have focused on the OBCFA content in milk from various ruminant species, the influences of different microorganisms on their content, and changes in the composition of the diet [6,8,9,10,11]. The objective of this study was to identify the factors that influence the content of OBCFAs in ruminant milk and determine which OBCFAs could serve as biomarkers for fermentation processes.

## 2. The Content of OBCFAs in Milk Fat

The global production of milk for human nutrition comes mainly from cows (about 85%), with lesser contributions from buffaloes (11%), goats (2.3%), sheep (1.4%), and camels (0.2%), while milk from other dairy species (e.g., donkeys, horses, and yaks) represents less than 0.1% [12]. Fat appears in milk as dispersed microscopic globules. Their degree of dispersion affects their digestibility, making milk fat the most easily absorbed animal fat. In 1 mL of milk, there are between 2 and 6 billion fat globules, most of which have diameters ranging from 2 to 4 µm [2,13,14]. Milk fat consists mainly of triacylglycerol (more than 95% of total lipids), with approximately 400 fatty acids (FAs) varying according to chain lengths, saturation levels, and stereospecific numbering (sn) positional distribution [15,16,17]. Milk fat is one of the most complex natural sources of fatty acids (FA), from C2 to C28, including even- and odd-numbered, saturated, monounsaturated, polyunsaturated cis and trans, linear and branched, and various keto- and hydroxy-FAs [5]. FAs are derived from body storage, dietary fatty acids, and the de novo synthesis of C4~C14 and partially C16 fatty acids, as well as the microbial metabolism in the rumen [9,13,17,18,19]. However, linear odd-chain FAs (C15:0 and C17:0) might be partially synthesized de novo in the mammary gland and animal tissues from propionate [6]. Cows in early lactation with a negative energy balance mobilize C16:0, C18:0, and *cis-*9 C18:1 from their body fat stores, increasing the concentrations of these FAs in their milk fat. This is also supported by previous studies that found that a negative energy balance and lipolysis resulted in less C5–C15 in milk fat and increased proportions of C16:0 and C18:0 in milk [20]. Short- and medium-chain milk FAs and OBCFAs in the early lactation period appeared to be negatively correlated with initial milk production and milk fat content [21]. OBCFAs with chain lengths of C17 carbon atoms decreased during the early lactation period, and similar changes have been observed for long-chain fatty acids [21]. It is possible to transpose fatty acids in non-esterified forms from adipose tissue to the mammary gland and subsequently re-esterify them with glicerol [22]. However, there are no papers that have described this process quantitatively. 

The fat composition of milk is crucial in terms of the quality and physical properties of dairy products [5,16,18], but also for animal health [16], as imbalances of certain nutrients can lead to changes in microbial populations and, consequently, these FAs [19]. Notably, the presence of odd- and branched-chain fatty acids occurs in milk due to microbial production and subsequent absorption by the mammary gland [23,24,25,26]. Therefore, the profiling of FAs in milk is of the utmost importance, as it can serve as a noninvasive biomarker for predicting the proportions of VFAs in the rumen, the synthesis of microbial proteins, or the susceptibility to SARA in dairy cows [24,25,26]. Additionally, the content of OBCFAs in dairy products is also critical to human health, as studies have confirmed its anti-tumor activity, its ability to decrease the incidence of necrotizing enterocolitis, type-2 diabetes, and cardiovascular diseases, and support for pancreatic *β*-cell function [12]. The content of OBCFA in milk fat depends on many factors, both external (e.g., feeding system, feed composition, ratio of roughage to concentrate, number of lactations, season, and geographical location) and internal (e.g., animal age, breed, lactation stage, and rumen pH) [16,25,27,28,29,30]. The individual FA concentrations in milk can be influenced by each of these factors and their interactions [27]. It has been proven that diet largely determines the content of OBCFAs in milk fat [31,32,33].

In general, the higher levels of OBCFAs in ruminants compared to non-ruminants are mainly due to the presence of rumen bacteria that synthesize these fatty acids, which are then incorporated into their cell membranes [19]. Among ruminants (Table 1), the content of OBCFAs varies from 3.33% (in goat’s milk) to 5.02% (in buffalo’s milk). Differences between species, and even within the same species, can be linked to variations in feeding and management practices, as well as environmental conditions. OBCFA concentrations can be influenced by the animal’s energy balance, the forage-to-concentrate ratio, and the presence of bioactive compounds in the feed. There is limited research in the literature on the effect of bioactive compounds on the concentration of OBCFAs in milk [19,34]. The incorporation of polyphenols into the diet of ruminants significantly modifies the rumen microbiome [35], which will affect the content of OBCFAs in milk.

The higher concentrations of OBCFAs in sheep’s milk are thought to be due to different de novo synthesis processes in their mammary glands (e.g., sheep use methylmalonyl-CoA more efficiently than cows or goats). In ruminants, the origin of *iso*- and *anteiso*-BCFAs differs, with the *iso* form derived from *iso*butyrate and *iso*valerate, while the *anteiso* form arises from 2-methylbutyrate. Biosynthetically, the last compounds originate from *iso*leucine. A decrease in the ratio of *iso* and *anteiso* FAs may result from an increased proportion of concentrates in the diet, which can lower the pH and alter the rumen bacterial population [19]. A study by Luo et al. [31] highlighted the complex dynamics of OBCFAs’ production in the rumen, their transport through the blood to the mammary gland, and their eventual presence in milk. The authors pointed to the significant potential contribution of adipose tissue and the de novo synthesis of milk fats in mammary tissue, emphasizing the need for further studies to determine the exact relative contributions of these sources to milk fat content [31]. The concentration of OBCFAs in dairy products is summarized in Table 2. It should be noted that there are significantly fewer studies in the literature on the content of specific OBCFAs in dairy products compared to milk, and these concentrations depend on the FA content of the raw material used.

Dairy farm feeding systems are influenced by economic, philosophical, and regulatory factors [44]. Manipulating cow diets can be an effective way to increase the fatty acid content in milk and dairy products [12,38], thereby contributing to the long-term health of consumers [13]. The impact of diet on the OBCFA content in cow’s milk is illustrated in Table 3.

The content of OBCFAs relative to fatty acids in milk ranges from 2.77 to 6.97 g·100 g^−1^ FA [5,45]. In the group of odd-chain fatty acids, pentadecanoic acid (C15:0) is the most prevalent, followed by heptadecanoic acid (C17:0). The most abundant branched-chain fatty acids in milk are *anteiso*-C15:0 and *anteiso*-C17:0, as well as *iso*-C15:0 and *iso*-C17:0 (Table 1).

The incorporation of fresh forage into dairy cow diets can improve the fatty acid (FA) profile of milk. However, the effects depend on the type of forage used [44]. For instance, a higher inclusion of corn silage in the diet results in a linear decrease in the concentration of C17:0 and a quadratic decrease in *anteiso*-C15:0 [46]. Zhang et al. [23] showed that different dietary ratios of forage to concentrate (F:C; 30:70, 50:50, and 70:30) influenced the OBCFA profiles. The highest concentrations of C11:0, C13:0, *iso*-C15:0, *iso*-C16:0, *iso*-C17:0, and C17:0 were observed in groups with an F:C ratio of 70:30, while the lowest concentrations of *anteiso*-C15:0, C15:0, and total OBCFAs were found in the group with a high-forage diet (differences were statistically significant. *p* < 0.05) [23]. Other studies have also confirmed the effect of diet on the composition of FAs in milk. Lopez et al. [13] also carried out a trial on two alpine farms. During winter, the first herd was fed a total mixed ration consisting of alfalfa, mixed grass hay, and concentrates, while during summer, the cows were fed a natural Alpine pasture. During winter, the second herd was fed a TMR consisting of meadow hay and concentrates, while in summer, during the day, the animals were fed a fresh grass cut daily, and in the evening, a TMR consisting of alfalfa hay and concentrates. Furthermore, on this farm, the cows were supplied with additional concentrates. The findings indicated that increasing the forage-to-concentrate ratio led to a higher proportion of *iso*-C14 and *iso*-C15 BCFAs in the cow’s milk [13]. The authors also noted that the concentrations of odd-chain fatty acids (C15:0, C17:0, and C17:1) were higher in the pasture-based cow’s milk and cheese, likely due to various types of pasture vegetation [13]. Increasing the starch content, forage digestibility, or legume proportion in the forage while reducing the forage-to-concentrate ratio or the neutral detergent fiber content promoted the growth of amylolytic bacteria and reduced cellulolytic bacteria [9]. By Vlaeminck et al. [6], it was found that diets rich in starch or with grass silage replaced by maize silage decreased *iso*-C14:0, *iso*-C15:0, and *iso*-C16:0 in milk fat.

The composition of milk fat is also affected by seasonal variations in feeding. The intake of fresh pasture (with or without supplementary feed) increases in spring and summer, while ensiled feed and concentrate are typical for winter [15,47]. Incorporating red clover silage into cow diets improves the proportions of OBCFAs in milk fat but reduces the amount of protein delivered to the cows by reducing the flow of crude microbial protein to the duodenum [48]. On the other hand, adding quebracho tannin extract to the diet decreases the individual proportions and yields of OBCFAs, except for *anteiso*-C15:0 and *anteiso*-C17:0 [49]. In a study by Moate et al. [49], the use of grape marc, a source of condensed tannins, reduced the proportions of C15:0 and C17:0 in milk fat. Jones et al. [50] found that condensed tannins from sainfoin inhibited the growth of *Butyrivibrio fibrisolvens*, but did not affect *Prevotella ruminicola*. Thus, such supplementation has no effect on the rumen-dominant *Prevotella* bacteria, which contain OBCFAs.

**Table 3:** The fatty acid content of cow’s milk depending on the diet (%).

Type of Diet	TMR	TMR + RSO	TMR	TMR + QTE_15_	TMR + QTE_30_	CS + GS	GS + H	GS-LO	GS-MD	GS-HI	GS100	GS67	GS33	GS0
Date of Feeding	–		October–February			June–September		April			October–December			
Breed	Holstein		German Holstein			Holstein		Swedish Red			Holstein-Friesian			
C11:0	0.080	0.061	–	–	–	–	–	–	–	–	–	–	–	–
C15:0	1.221	1.024	1.02	0.96	0.92	0.928	0.914	0.94	1.00	1.06	1.23	1.05	0.93	0.91
C17:0	0.480	0.428	0.50	0.49	0.47	0.479	0.500	0.53	0.57	0.67	0.70	0.66	0.59	0.52
*iso*C13:0	0.031	0.026	–	–	–	–	–	–	–	–	–	–	–	–
*anteiso*C13:0	0.022	0.016	–	–	–	–	–	–	–	–	–	–	–	–
*iso*C14:0	0.136	0.147	0.064	0.058	0.054	0.059	0.074	0.07	0.07	0.08	0.09	0.08	0.08	0.09
*iso*C15:0	0.190	0.166	0.20	0.18	0.18	0.172	0.187	0.18	0.19	0.21	0.30	0.24	0.24	0.21
*anteiso*C15:0	0.817	0.750	0.45	0.42	0.45	0.369	0.407	0.39	0.40	0.42	0.45	0.39	0.39	0.41
*iso*C16:0	0.351	0.244	0.17	0.16	0.16	0.169	0.188	0.26	0.29	0.26	0.16	0.15	0.18	0.19
*iso*C17:0	0.112	0.127	0.30	0.29	0.29	0.300	0.331	0.33	0.29	0.47	0.38	0.37	0.37	0.37
*anteiso*C17:0	0.334	0.285	0.43	0.42	0.44	–	–	–	–	–	0.41	0.40	0.40	0.42
OBCFA	4.116	3.683	35.5	33.8	32.7	–	–	2.77	2.85	3.25	–	–	–	–
Reference	[2]		[51]			[21]		[45]			[46]			

Abbreviations: RSO—soybean oil; QTE—quebracho tannin extract supplemented at levels of 15 g and 30 g·kg^−1^ DM; CS + GS—corn silage and grass silage (600:400, wt/wt on a DM basis); GS + H—grass silage and hay (350:650, wt/wt on a DM basis); Grass silage (at levels of 50 (LO), 70 (MD), and 85% (HI) of total DM intake) and grain based concentrate. Treatments had a roughage-to-concentrate ratio of 80:20 (on a DM basis). Concentrate was similar for all diets. Roughage consisted of (all DM basis); 100% grass silage (GS100); 67% grass silage and 33% corn silage (GS67); 33% grass silage and 67% corn silage (GS33); 100% corn silage (GS0).

The fatty acid content in cow milk fat, including odd- and branched-chain fatty acids (OBCFA), shows variation depending on the stage of lactation (Table 4) [21,52,53]. The digestion of lactating dairy cows largely depends on the microbial population in the rumen [54]. At the beginning of lactation, cows generally mobilize adipose fatty acids, some of which are incorporated into milk fat. A higher intake of long-chain fatty acids (LCFAs) during this period reduces the proportions of short- and medium-chain fatty acids (SMCFAs) in milk fat, due to both dilution effects and the inhibition of de novo FA synthesis. Thus, SMCFA ratios are comparatively low in the early stages of lactation and rise until at least 8–10 weeks, while LCFA ratios gradually decline. Although changes in SMCFAs and LCFAs in relation to the stage of lactation are well-documented, there are few reports on the effect of lactation stage on the OBCFA content [21].

The contents of *iso* and *anteiso* fatty acids in milk fat are significantly lower (*p* < 0.05) in the early stage of lactation compared to the mid or late stage [10]. However, the C17:0 level is higher in the early stage of lactation than in the later stage. The content and composition of milk fat are also influenced by physiological and metabolic changes occurring in cows. The number of births also plays a role. Cows undergoing their first or second calving have a higher odd- and branched-chain fatty acid content [10].

In the study conducted by Bainbridge et al. [53], a comparison was made between the content and profile of fatty acids in the milk of Holstein (HO), Jersey (JE), and the first generation of Holstein–Jersey crossbreeds (CB) during lactation. The animals were fed a total mixed ration (TMR) with a forage-to-concentrate ratio of 70:30. The authors found that the total OBCFA content was higher in the Jersey than in the Holstein and crossbreed cows at 185 and 275 days in milk (DIM). The concentration of individual branched-chain fatty acids also varied, with *iso*-C14:0 and *iso*-C16:0 being higher in the Jersey milk compared to that of the Holsteins and crossbreeds from 95 to 275 DIM [53].

## 3. The Synthesis of OBCFAs in Cow’s Milk Fat

The source of fatty acids in ruminant milk fat includes those generated during rumen processes, lipids from the diet, and mobilized body fat [17]. The OBCFAs in ruminant milk and meat generally consist of odd- and branched-chain fatty acids containing from 13 to 20 carbon atoms. The OBCFA content in milk fat typically represents 2–3% of all fatty acids, deriving from rumen microbial cell membranes and endogenous synthesis in the mammary gland [6,45].

In rumen processes, odd-chain fatty acids like C15:0 and C17:0 are formed during elongation from propionate or valerate [56]. These fatty acids are synthesized using acetyl-CoA as a starter through the repeated condensation of malonyl-CoA derived from the modification of various fatty acids. The precursors of branched-chain fatty acids (*iso* and *anteiso*) are branched-chain amino acids: valine, leucine, *iso*leucine, or short-chain branched carboxylic acids, including *iso*valeric acid, *iso*butyric acid, and 2-methylbutyric acid [3,6]. The de novo synthesis of C15:0 and C17:0 also occurs in ruminant mammary gland tissues and adipose tissue from propionate. This conclusion arises from comparisons of the levels of these fatty acids in milk fat with duodenal flow, which is lower than the observed levels in milk. The importance of Δ-9 desaturase in the conversion of C17:0 to *cis*-9 C17:1 in mammary tissues is also emphasized [57]. However, this does not apply to all OBCFAs, as the effects differ depending on the branching (*iso* or *anteiso*). Linear odd-chain fatty acids are only partially synthesized in the mammary gland, regardless of whether they are *iso* or *anteiso* [57,58,59].

Strategies to increase the OBCFA content in milk, particularly the *iso* acids, focus on increasing forage in the diet [45]. The OBCFA concentration in milk correlates more strongly with acetic acid than with propionates and butyrate [60]. Depending on the acid undergoing biohydrogenation, the stages of this process differ. For years, it was believed that *Butyrivibrio fibrisolvens* was the only bacterium capable of biohydrogenation. However, many other bacteria are now known to contribute to this process [55,57]. Feeding cows a high amount of concentrate or starch produces more propionate, which appears to inhibit cellulolytic bacteria, leading to changes in the milk’s OBCFA profile through reduced levels of *iso*-C14:0, *iso*-C15:0, and *iso*-C16:0 in milk fat [6].

The effect of fat supplements on fermentation processes and the biohydrogenation of fatty acids is well documented. The addition of C18:2 n-6 (e.g., sunflower oil), C18:3 n-3 (e.g., flaxseed oil), or C20:5 n-3 and C22:6 n-3 (e.g., fish oil or marine algae) to cows’ feed significantly impacts the rumen microflora [61,62,63]. Gram-negative bacteria are less sensitive to fat supplements compared to Gram-positive ones. These supplements lead to increased propionate production, but they might also inhibit the endogenous synthesis of certain BCFAs, such as *iso*-C14:0 and *iso*-C16:0 [2,64]. Supplementation with PUFA (e.g., marine algae) in the form of meal can increase the *iso*-C17:0 content in milk fat while reducing *iso*-C15:0 [65].

Additionally, fat supplementation in the diet of lactating cows is a strategy used to increase the energy density of the feed ration. In cows during early lactation, this method reduces the severity of negative energy balance, which can support milk production. However, lipid supplementation can impact various metabolic processes that influence the fatty acid profile of milk. For example, the addition of lipids to feed can change the rumen environment and affect microbial fermentation and volatile fatty acid production. For example, *cis*-9,*cis*-12 C18:2 (a soybean oil fatty acid) has been shown to have toxic effects on the rumen microbiota, especially cellulolytic bacteria [2].

## 4. OBCFAs as a Biomarker of Rumen Fermentation

Modern chromatographic techniques enable the analysis of the fatty acid profile of ruminant milk, allowing for the identification of a group of about a dozen OBCFAs. The most commonly used technique for the quantitative and qualitative identification of odd-chain fatty acids, as well as iso and *anteiso* isomers, is gas chromatography with an FID or MS detector of ester derivatives (FAME methyl esters or FAEE ethyl esters) obtained after lipid hydrolysis or pure acids (using dedicated columns). For odd-carbon acids, there are a number of commercially available analytical standards (including 37 FAME mixtures). Unfortunately, for *iso* and *anteiso* acid isomers, the availability of analytical standards is limited. The solution to this is to perform the gas chromatography of FAME or FAEE with the calculation of retention indices and a comparison of the obtained values with data present in databases, for example, NIST23 or Mondello [66]. For most of the columns used, it is easy to compare the determined indexes with the data from the literature. A complementary and unambiguous method for identifying branched acids is to perform analysis by the EI-MS/MS technique for ion-trap MS detectors. Ran-Ressler presented MS/MS decompositions of *iso* and *anteiso* isomers for all acids from C-12 to C-31, enabling their unambiguous qualitative identification [43]. LC-MS techniques have also been developed in recent years to enable the detection of OBCFAs by utilizing the available standards using a C-18 column and a Chiralpak IG-U column (with better selectivity). The feasibility of identifying these isomers using the ESI-MS/MS technique was demonstrated by [67]. This method offers a promising and non-invasive way to evaluate the fermentation processes occurring in the rumen. Analyzing milk composition can be extremely useful for the prevention of metabolic disorders in cows, such as subacute ruminal acidosis, which is fairly common in high-producing herds [68]. Below is an up-to-date review of studies on the role and significance of OBCFAs in the fermentation processes taking place in the rumen of ruminants and the implications of these relationships for diagnosing metabolic disorders in dairy cows.

The content of OBCFAs in the fat of ruminant milk may reflect the composition of the rumen microbiota. Cellulolytic bacteria contain high amounts of *iso* fatty acids, including *Ruminococcus flavefaciens*, *Ruminococcus albus*, and *Butyrivibrio fibrisolvens*. *Prevotella* strains contain *anteiso*-C15:0 and *anteiso*-C17:0 [69]. The proportion of certain branched fatty acids (*iso*-C13:0, *anteiso*-C15:0, *iso*-C14:0, *anteiso*-C15:0, and *iso*-C16:0) is higher in bacteria compared to protozoa, while protozoa tend to have higher proportions of *iso*-C17:0 and *anteiso*-C17:0 [70]. There is a relationship between the metabolic processes of rumen bacteria and the ratios of short-chain fatty acids: acetic, propionic, and butyric acids [6]. The concentration of *iso*-C16:0 in the rumen content could be a potential marker for assessing the total amount of volatile fatty acids [25]. According to Vlaeminck et al. [71], the OBCFAs in milk fat can serve as a pattern for the fermentation processes occurring in the rumen. This is due to the correlation between the molar ratio of volatile fatty acids in the rumen and the content of individual odd-chain and branched-chain fatty acids in milk fat. The concentration of propionate in the rumen was positively correlated with C15:0 and C17:0 in milk fat, while the concentration of acetate in the rumen was negatively correlated with these acids, but positively correlated with *iso*-C14:0 and *iso*-C16:0 [9]. More recent studies highlight the role of milk *iso* FAs as biomarkers for dietary fiber concentration [24]. Other research indicates that, while the proportion of milk OBCFAs is positively correlated with protozoa of the genus *Isotricha*, the proportion of C17:0 in milk was negatively correlated with *Butyrivibrio* [28].

In general, cellulolytic bacteria (e.g., *Ruminobacter amylophilus*. *Selenomonas ruminantium*, *Streptococcus bovis*, and *Succinimonas amylolytica*) are characterized by low concentrations of *iso* branched-chain fatty acids [9,72]. The abundance of *Ruminococcus albus*, *Ruminococcus flavefaciens,* and *Eubacterium ruminantium* was significantly correlated with the C13:0 content in milk, suggesting that the C13:0 levels in milk could reflect the population of cellulolytic bacteria in the rumen content [25]. The conversion of *iso*-C15:0 to *iso*-C17:0 might occur with the involvement of *Fibrobacter succinogenes* [55]. The OBCFA profile of *Butyrivibrio* spp. strains is much more heterogeneous. They break down a wide range of substrates, including fiber, starch, and fatty acids, and produce large amounts of lactate and acetate [73]. Generally, fibrolitic bacteria are rich in *iso*-OBCFAs, while amylolytic bacteria contain more *anteiso*-OBCFAs [8]. Cellulolytic bacteria had stronger correlations with OBCFA concentrations compared to amylolytic bacteria [25]. *Butyrivibrio fibrisolvens* play a key role in linking the synthesis of long-chain and short-chain fatty acids [6]. *Selenomonas ruminantium* is highly correlated with the total concentration of OBCFAs and branched-chain fatty acids in milk, indicating that changes in the OBCFA concentration in milk are involved in the interactions among various rumen microorganisms [55].

The occurrence of subacute ruminal acidosis (SARA) in high-producing cow herds is a significant problem in all intensive production systems. It is common practice to feed high-yielding dairy cows with large amounts of starch and a low fiber content to meet the energy demands of milk production [74]. Such unbalanced diets may negatively impact rumen function due to high acid production (including lactic acid) and a reduced buffering capacity. Cows with a low rumen pH for prolonged periods differed in the number and composition of rumen bacteria from those that barely experienced pH drops below normal values [75]. There was an increased abundance of lactate producers, such as *Streptococcus Sharpea* and *Succinivibrionaceae*, as well as starch degraders from the genera *Prevotellaceae*, *Ruminococcus,* and *Ruminococcaceae* in the rumen bacteria of cows highly susceptible to SARA [75]. The SARA diet significantly reduces the diversity of the rumen microbial community [76]. In contrast, fiber-degrading species, such as *Ruminococcus albus*, *Ruminococcus flavefaciens*, and *Butyrivibrio fibrisolvens*, exhibit high proportions of *iso* fatty acids [6,71]. The functional profile of the ruminal microbiome corresponded to known metabolites impacted by high concentrate feeding during experimentally induced SARA [77]. As demonstrated by these studies, the dynamics of microbiome changes in acidosis occur and the population of key bacteria (e.g., genus *Bacteroidetes* or *Prevotella*) can also increase, simultaneously leading to a change in the profile of the produced fatty acids. Furthermore, in bacterial lipopolysaccharides (LPS), a component of the cell wall of Gram-negative bacteria causes defects in the barrier function of the epithelium and triggers acute phase responses and epithelial inflammation [76,78].

The use of excessive amounts of concentrate feed and a shift in the microbial population that results in an increase in *Streptococcus bovis* can initiate a chain of events that may ultimately lead to subacute ruminal acidosis (SARA). Diets high in fiber tend to lower the concentration of odd- and branched-chain fatty acids (OBCFA) in milk [79]. Some OBCFA are identified as markers for the early recognition of ruminal acidosis. An increase in milk C17:0 and C17:1 *cis*-9 and a decrease in *iso*-C14:0 suggest subacute acidosis [9]. Therefore, the rise in these fatty acids in cow’s milk can occur before clinical signs of acidosis appear, which is confirmed by recent studies by Sandri et al. [80]. Odd-chain fatty acids increased, whereas branched-chain fatty acids were reduced during SARA induction [80]. An increase in milk *trans*-10 C18:1 has greater potential as an indicator of acute ruminal acidosis, while C15:0 and C17:0 may be markers for SARA. Other studies indicate that besides *trans*-10 C18:1, other fatty acids (*iso*-C13:0, *iso*-C16:0, and *cis*-9, *trans*-11 C18:2) might also be useful in studies on cow acidosis [29]. From a practical perspective, monitoring rumen pH seems simpler and more useful for field diagnostics. Additionally, Westreicher-Kristen et al. [48] demonstrated a correlation between the OBCFA profile and the supply of microbial crude protein (MCP) in lactating Holstein cows. Total urinary purine derivative (PD) excretion is correlated with an increase in the concentration of *iso*-C15:0, *anteiso*-C17:0, and *iso*-C16:0 in milk lipids.

Under normal rumen conditions, *iso*-C15:0 is particularly abundant in cellulolytic populations [9]. Cows with SARA are characterized by a higher proportion of *trans*-10 C18:1, C15:0, and the *trans*-10 C18:1 to *trans*-11 C18:1 ratio in milk fat [81]. By contrast, the concentration of *trans*-11 C18:1 remains stable. Previously, it was suggested that high milk proportions of C15 and C17 odd-chain and *anteiso* fatty acids could be used as biomarkers of ruminal acidosis, indicating an increased dietary starch intake [9]. In the case of long-term subclinical ruminal acidosis, the percentage of fat in milk was less correlated with pH-related variables (such as time with pH <5.6 or <6.0, and minimum pH) and acetate production than with *iso*-C14:0 and *iso*-C15:0 [29]. In these studies, PCA analysis showed the greatest differences in *iso*-C14:0 and *trans*-10 C18:1 concentrations between SARA-affected cows and healthy ones. On the other hand, biohydrogenation intermediates previously associated with rumen acidosis (such as *trans*-10 C18:1 and *trans*-10, *cis*-12 C18:2) were not selected as the most discriminating milk fatty acids in this study. The most effective predictors in milk fatty acids for a low rumen pH were C18:2 *cis*-9, *trans*-11, *iso*-C16:0, and *iso*-C13:0 [29]. The determination of VFA and OBCFA in feces from cows subjected to SARA revealed a slight reduction in the proportion of *iso*-OBCFAs, especially *iso*-C15:0 and *iso*-C16:0 [82]. Greater significance in warning about the emergence of SARA after calving may lie with the analysis of fecal bacterial communities in the prepartum period.

The PCA analysis conducted by Toral et al. [24] points to significant correlations between certain OBCFAs and dietary fiber or starch, which aligns with the potential role of these fatty acids as biomarkers of cellulolytic and amylolytic bacteria in the rumen. However, there are some limitations to using OBCFA content as a marker of rumen function. Vlaeminck et al. [59] demonstrated higher concentrations of C15:0, *iso*-C17:0, *anteiso*-C17:0, and *cis*-9–C17:1 in milk samples compared to those from the duodenum. This suggests the occurrence of de novo synthesis involving the desaturation and elongation of these fatty acids [59]. Prado et al. [33] also suggest that concentrations of C15:0, C17:0, *iso*-C15:0, *iso*-C17:0, *anteiso*-C15:0, and *anteiso*-C17:0 were influenced by the concentrations in the duodenum, indicating the synthesis of these fatty acids from C3 units for linear-chain odd FAs, and from C2 units for branched-chain FAs.

In contrast to the above studies, a meta-analysis conducted on sheep questions the association between these milk fatty acids and dietary starch, casting doubt on the prediction of acidosis considering *anteiso*-15:0 and linear odd-chain FAs [32]. Additionally, there was no effect of dietary starch, lipids, or ADF on the contents of *iso*-13:0, *iso*-14:0, and *iso*-15:0 according to Gómez-Cortés et al. [83].

Both sodium acetate and sodium bicarbonate increase milk fat production, but only sodium bicarbonate added to cow diets raises OBCFA levels [32]. Vazirigohar et al. [60] suggest that the usefulness of OBCFA concentrations in milk to predict the ruminal molar proportion of acetate is stronger than that for propionate or butyrate in diets containing supplemental fats for lactating cows. Cows’ diets supplemented with fatty acids containing 18:2n-6 increase the milk fat content of branched-chain FA slike *iso*-13:0, *iso*-14:0, *iso*-15:0, *iso*-16:0, *iso*-17:0, *anteiso*-13:0, and *anteiso*-15:0 [60]. In contrast, the use of protein in the form of extruded soybean meal resulted in proportional decreases in C16 and OBCFAs [84].

Subclinical ketosis is a common condition during the peripartum period and early lactation in high-yielding cows. It has a significant impact on cows’ health and productivity. Subclinical ketosis occurs when blood concentrations of *β*-hydroxybutyric acid exceed 1.4 mmol·L^−1^. These cows experienced a reduction in milk yield of 2.4 kg per day in the two-week period following diagnosis [85]. It was shown that 70% of cows with hyperketonemia had a high ratio of *cis*-9 C18:1 to C15:0 in milk fat, which in practice would require the analysis of these two fatty acids [86]. Additionally, higher *anteiso*-C17:0 levels in cows with ketosis reflect greater body fat mobilization in the pathogenesis of ketosis. *Cis*-9 C18:1 is the dominant fatty acid in ruminant adipose tissue, and its increased milk content can be used as a marker for lipomobilisation and/or negative energy balance [87]. OBCFA <C16 reflects rumen synthesis modifications, incorporation into milk fat, and de novo synthesis by the mammary gland, in contrast to long-chain FA [59]. Pires et al. [88] indicate that OBCFA >C16 is associated with body fat mobilization. Experimentally, early-lactation cows on a restricted diet showed high correlations of ∑ C6:0 to C15:0, C18:0, *cis*-9 C18:1, and ∑ OBCFA >C16 in milk with metabolic status and lipomobilization [88]. Therefore, milk ∑ OBCFA >C16 concentration is related to body fat mobilization and is an important marker of energy balance in cows. Acetate supplementation increases milk fat yield and plasma acetate and β-hydroxybutyrate concentrations, which are major metabolic substrates for mammary lipogenesis [79]. On the other hand, acetate supplementation tended to decrease the yield of odd and branched-chain FA [79]. The authors of this study suggest that acetate supplementation provides more even and straight carbon substrate for mammary and ruminal de novo lipogenesis and likely competes with OBCFA carbon substrates in de novo synthesis both in the rumen and mammary gland.

Milk fatty acid profile can also be used as an indicator to estimate methane emissions [89]. *Iso* forms of OBCFA have been linked to methane reduction and used to predict methane emissions. Both C15:0 and C17:0 were positively correlated with CH_4_ [90,91,92]. It has been shown that OBCFAs produced by rumen microbial activity, such as *iso*-C14, *iso*-C15, *iso*-C16, and C23:0, are reduced by a specific anti-methanogenic additive that lowers enteric methane production [93]. Furthermore, the concentration of VFA in the rumen can be related to methane emissions, indicating that analyzing OBCFA in ruminant milk could help determine CH_4_ emissions [9], which can be used in the future to reduce the negative impact of cattle farming on the environment.

## 5. Conclusions

The content of OBCFAs in cow milk fat is largely a result of bacterial processes in the rumen, although endogenous synthesis and/or conversion of certain fatty acids due to lipomobilization also play a role. The concentration of OBCFAs in cow milk fat depends on various factors, including the composition of the feed ration (the proportion of roughage and concentrate), rumen content pH, and ammonia concentration. More research is needed to resolve discrepancies in OBCFAs content and correlations depending on various factors to optimize the effectiveness of approaches aimed at increasing the OBCFA content in milk and dairy products, thereby improving the quality of animal and human health. The analysis of the OBCFAs profile is a promising, non-invasive method for assessing fermentation processes in the rumen. Certain OBCFAs in milk fat could be markers for rumen acidosis and microbial protein flow into the duodenum. OBCFAs are also among the fatty acids linked to body fat mobilization. However, further research is needed to better describe the transfer of OBCFA from the rumen to milk to improve the accuracy of predicting rumen changes or preventing metabolic disorders. Moreover, analyzing OBCFAs in ruminant milk can help determine CH_4_ emissions.

## Figures and Tables

**Table 1:** The OBCFAs content in milk lipids depending on the ruminant species (%).

FA	Milk								
	Buffalo		Cow			Goat		Sheep	
	[19]	[36]	[19]	[37]	[38]	[19]	[11]	[19]	[8]
C11:0	0.02	0.06	0.09	0.06	0.08	0.19	–	0.29	0.1
C15:0	1.15	1.34	1.06	1.18	1.05	0.75	–	1.18	1.26
C17:0	0.51	0.54	0.46	0.54	0.46	0.79	–	0.76	0.65
*iso-*C13:0	0.02	0.07	0.09	0.06	0.08	0.03	0.01	0.03	0.06
*anteiso-*C13:0	0.04	–	–	–	–	0.02	–	0.04	0.01
*iso-*C14:0	0.19	0.26	0.12	0.17	0.11	0.1	0.06	0.12	0.1
*iso-*C15:0	0.32	0.4	0.22	0.28	0.21	0.19	0.16	0.29	0.2
*anteiso-*C15:0	0.54	0.64	0.44	0.53	45	0.33	0.32	0.56	0.49
*iso-*C16:0	0.39	0.46	0.233	0.32	0.22	0.25	0.19	0.33	0.29
*iso-*C17:0	0.24	0.3	0.25	0.32	0.26	0.31	0.42	0.42	0.36
*anteiso-*C17:0	0.37	0.41	0.42	0.42	0.4	0.39	0.44	0.5	0.5
BCFA	2.1	2.65	1.78	2.08	1.74	1.6	–	2.29	2.01
OBCFA	3.78	5.02	3.38	3.97	3.64	3.33	–	4.51	3.88
Σ *iso*-FA/Σ BCFA, %	55.19	56.22	51.66	55.29	50.57	53.93	53.88	51.75	50.25
Σ *anteiso*-FA/Σ BCFA, %	44.81	39.62	48.34	45.67	48.85	46.07	46.12	48.25	49.75

**Table 2:** The OBCFAs content in various dairy products (%).

FA	Cheese								Yoghurt	Butter	Sour Cream	Ice Cream
	Cow						Goat	Sheep				
	[39] ^1^	[40] ^2^			[41] ^3^		[42]	[42]	[43]	[43]	[43]	[43]
		P-UL	H-UL	MS-LL	0	60						
C11:0	0.32–0.39	–	–	–	0.02	0.02	–	–	–	–	–	–
C15:0	1.12–1.20	1.23	1.24	1.28	0.23	0.27	0.89	1.21	–	–	–	–
C17:0	0.27–0.62	0.62	0.57	0.61	0.16	0.18	0.75	0.66	–	–	–	–
*iso-*C13:0	0.11–0.13	–	–	–	0.02	0.02	0.02	0.02	–	–	–	–
*anteiso-*C13:0	0.10–0.16	–	–	–	0.02	0.03	0.07	0.05	–	–	–	–
*iso-*C14:0	0.25–0.29	–	–	–	0.03	0.03	0.11	0.1	0.12	0.17	0.05	0.14
*iso-*C15:0	1.07–1.28	0.38	0.31	0.31	0.05	0.06	0.24	0.28	0.15	0.01	0.11	0.33
*anteiso-*C15:0	0.48–0.53	0.66	0.62	0.59	0.11	0.12	0.39	0.55	0.62	0.63	0.46	0.42
*iso-*C16:0	0.15–0.16	0.46	0.34	0.34	0.07	0.07	0.25	0.29	0.29	0.34	0.24	0.46
*iso-*C17:0	–	0.46	0.42	0.38	0.07	0.08	0.17	0.41	0.25	0.31	0.3	0.17
*anteiso-*C17:0	0.37–0.45	0.42	0.39	0.38	0.12	0.14	0.4	0.5	0.59	0.38	0.36	0.57
OBCFA	4.74–5.39	4.53	4.13	4.12	0.98	1.13	3.12	4.2	–	–	–	–

^1^ Range of FAs content of eight Camembert-type cheese purchased at supermarkets by authors; ^2^ P-UL—pasture-based-upland; H-UL—hay-based-upland; MS-LL—maize silage-based-lowland; ^3^ 0, 60—days of dietary supplementation with humic-mineral substances in cows’ diet.

**Table 4:** The fatty acid content (g·100 g^−1^) of cow’s milk depending on the stage of lactation feeding TMR diet.

FA	Early					Middle					Late				
	HO	HO *	HO	JE	CB	HO	HO *	HO	JE	CB	HO	HO *	HO	JE	CB
C11:0	0.03	0.08	0.21	0.24	0.25	0.03	0.07	0.26	0.28	0.28	0.04	0.05	0.26	0.28	0.30
C15:0	0.84	1.12	1.21	1.19	1.30	0.84	1.01	1.24	1.20	1.16	0.78	0.94	1.13	1.21	1.20
C17:0	0.57	0.51	0.68	0.65	0.72	0.43	0.50	0.67	0.65	0.65	0.47	0.51	0.62	0.65	0.70
*iso*C13:0	–	0.05	0.02	0.02	0.02	–	0.06	0.02	0.02	0.02	–	0.06	0.04	0.03	0.05
*anteiso*C13:0	–	0.04	0.05	0.08	0.07	–	0.05	0.10	0.09	0.09	–	0.05	0.09	0.10	0.11
*iso*C14:0	–	0.15	0.07	0.12	0.12	–	0.15	0.10	0.12	0.12	–	0.16	0.13	0.13	0.16
*iso*C15:0	0.14	0.19	0.19	0.18	0.20	0.15	0.20	0.19	0.18	0.19	0.15	0.27	0.21	0.22	0.23
*anteiso*C15:0	0.38	–	0.42	0.36	0.42	0.39	–	0.43	0.37	0.40	0.38	–	0.43	0.42	0.48
*iso*C16:0	0.25	0.36	0.19	0.26	0.25	0.19	0.32	0.26	0.32	0.26	0.16	0.32	0.30	0.35	0.39
*iso*C17:0	0.44	0.54	0.30	0.24	0.30	0.52	0.55	0.26	0.23	0.26	0.55	0.60	0.26	0.23	0.30
*anteiso*C17:0	0.83	0.46	0.09	0.10	0.10	1.13	0.43	0.14	0.11	0.09	1.01	0.44	0.06	0.07	0.09
OBCFA	3.54	3.99	5.10	5.05	5.59	3.57	3.82	5.51	5.23	5.16	3.69	3.89	5.16	5.38	5.80
Reference	[55]	[10]	[53]			[55]	[10]	[53]			[55]	[10]	[53]		

Abbreviations: HO—Holstein, JE—Jersey, CB—HO × JE crossbreed; *—expressed from proportion of corresponding methyl esters.

## Data Availability

Not applicable.
